# Hierarchical Mechanistic Modeling of Complex Toxicity Endpoints from Public Concentration–Response Data

**DOI:** 10.1021/acs.est.5c09326

**Published:** 2026-01-13

**Authors:** Elena Chung, Daniel P. Russo, Lauren M. Aleksunes, Genoa R. Warner, Hao Zhu

**Affiliations:** † Department of Chemistry and Biochemistry, 3536 Rowan University, Glassboro, New Jersey 08028, United States; ‡ Center for Biomedical Informatics and Genomics, School of Medicine, Tulane University, New Orleans, Louisiana 70112, United States; § Department of Pharmacology and Toxicology, Ernest Mario School of Pharmacy, 43337 Rutgers University, Piscataway, New Jersey 08854, United States; ∥ Department of Chemistry and Environmental Science, College of Science and Liberal Arts, 5965 New Jersey Institute of Technology, Newark, New Jersey 07103, United States

**Keywords:** new approach methodologies, hierarchical modeling, toxicity prediction, high-throughput screening, adverse outcome pathways, artificial intelligence, big data

## Abstract

High-throughput screening (HTS) programs have generated abundant data on numerous chemicals, supporting the discovery of toxicity mechanisms and advancing understanding of adverse outcome pathways (AOPs) in chemical toxicity. However, organizing and interpreting these data for predictive modeling remain challenging due to inconsistent repository formats, varied program objectives, heterogeneous assay targets, and differences in experimental protocols, including concentration ranges. To address these limitations, we developed a hierarchical mechanistic modeling framework that systematically structures and interprets concentration response HTS data. The model integrated curated data sets by mapping metadata from 455 PubChem assays to 216 protein targets and 103 biological pathways in WikiPathways. Assay-level concentration–response data were organized within a biologically layered hierarchy to construct AOP-based models. The resulting models generated pathway-level toxicity scores that quantified compound potency by integrating inferred protein activity and downstream pathway perturbations. In total, 103 pathways were statistically associated with five *in vivo* toxicity endpoints: acute systemic, maternal, developmental, human hepatotoxicity, and preclinical hepatotoxicity. This hierarchical framework leverages HTS metadata to predict diverse *in vivo* toxicity outcomes and enhance mechanistic interpretability of pathway-level effects. It links chemical bioactivity to adverse outcomes, providing a quantitative basis for compound ranking, potency assessment, and hazard prediction. Overall, this framework offers a structured, scalable method for integrating large bioactivity data sets into computational toxicology, supporting chemical risk assessment and early-stage drug discovery.

## Graphical abstract

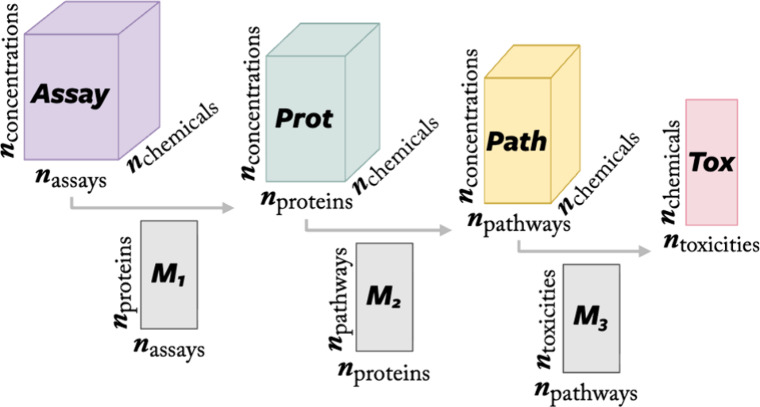

## Introduction

Emerging environmental contaminants, including industrial chemicals, pharmaceuticals, personal care products, and pesticides, pose substantial risks to human and ecological health through environmental persistence, propensity for bioaccumulation, and the potential to induce adverse biological effects.^1–3^ In 2019, hazardous chemical exposure was estimated to contribute to approximately 2 million deaths (3.6% of total global mortality) and 53 million disability-adjusted life years (2.1% of total DALYs), constituting a significant public health burden.^4^ These chemicals can accumulate in human tissues, contributing to acute toxicity following short-term exposure and chronic health outcomes such as developmental and hepatic injury.^5^ For example, endocrine-disrupting chemicals modulate hormone-regulated signaling pathways (e.g., thyroid, estrogen, and androgen receptors), triggering reproductive, developmental, and metabolic disorders.^6^ Despite ubiquitous human exposure to thousands of synthetic and naturally occurring chemicals, the toxicological profiles of many chemicals remain uncharacterized, complicating regulatory evaluation and chemical safety assessment.^7^

Traditionally, *in vivo* toxicity testing has guided chemical risk assessment. Common endpoint metrics include the median lethal dose (LD_50_), which estimates the dose expected to cause mortality in 50% of a test population and is derived from acute systemic toxicity tests. Similarly, the no-observed-adverse-effect level (NOAEL) identifies the highest dose in repeated-dose studies (e.g., subchronic or chronic exposure experiments) where no treatment-related adverse effects are observed.^8,9^ Although foundational to toxicological evaluations, these approaches are often resource-intensive, time-consuming, costly, and impractical for assessing the extensive number of untested chemicals. These limitations have prompted sustained federal support for new approach methodologies (NAMs) and more recently, regulatory communication from the United States Food and Drug Administration (US FDA), Environmental Protection Agency (EPA), and National Institutes of Health (NIH), to accelerate the adoption of nonanimal, human-relevant models.^10–13^ Such NAMs include high-throughput screening (HTS), *in silico* modeling approaches, and mechanistic frameworks like the adverse outcome pathway (AOP), all of which can support chemical risk assessment more efficiently.^14^

Large-scale screening initiatives such as the EPA Toxicity Forecaster (ToxCast) and the Toxicology in the 21st Century (Tox21) programs have generated substantial HTS data relevant to chemical toxicity.^15^ Conducted in collaboration with the FDA, the National Center for Advancing Translational Sciences (NCATS), and the National Toxicology Program (NTP), these initiatives have produced extensive, publicly available HTS data sets suitable for systemic toxicity evaluation and chemical prioritization.^16,17^ HTS methods can rapidly test thousands to millions of chemicals against diverse molecular targets and signaling pathways, offering complementary mechanistic data for integration within the AOP framework.^18,19^ Endorsed by the Organisation for Economic Co-operation and Development (OECD), the AOP framework describes the biological progression of toxicity from molecular initiating events (MIEs), in which a stressor directly interacts with a molecular target to trigger a biological response pathway, through key events (KEs), to adverse outcomes (AOs) such as chemical-induced hepatotoxicity.^20–22^ However, translating HTS-derived data into reliable *in vivo* toxicity predictions remains challenging due to the complexity of biological systems and limited understanding of upstream and downstream signaling cascades.^23^

Computational modeling approaches have sought to integrate large-scale HTS data into predictive toxicology frameworks to overcome these AOP limitations in the current era of big data.^24–26^ Traditional modeling approaches, such as quantitative structure–activity relationships (QSARs), predict toxicity based on chemical structure and physicochemical properties.^24,27^ These models, however, are often limited by narrow chemical space coverage, sparse training data, and constrained capacity to accommodate heterogeneous large-scale data sets.^28^ Advances in machine learning approaches improved HTS-based predictive toxicity modeling by incorporating concentration–response data and exposure-relevant metrics.^29–33^ Prior supervised learning methods identified toxicity biomarkers, and hybrid QSAR models have enhanced predictive accuracy for endpoints with limited data.^25,34^ Nonetheless, harmonizing and extracting mechanistic information from HTS data remains a central challenge, necessitating new modeling strategies to process, curate, and structure concentration–response data into biologically meaningful toxicity mechanisms.

Hierarchical modeling provides a robust strategy for organizing heterogeneous data into multilevel biological representations for toxicity modeling.^35^ Prior efforts to build hierarchical models include dual-layer QSAR models for acute toxicity prediction^36,37^ and the classification of cytochrome P450 (CYP) interactions using multidimensional data sets.^38^ Recent developments in hierarchical deep learning extended these applications by embedding pathway-informed representations (e.g., using transfer learning and unsupervised learning) to handle incomplete or inconsistent inputs and generate interpretable outputs.^39–41^ Structured modeling approaches like PROGENy, have projected high-dimensional transcriptomic data onto pathway-level activity via matrix-based transformation, enhancing mechanistic resolution.^42^ Nevertheless, most existing modeling studies rely on single-source data sets and do not fully leverage concentration–response HTS data from diverse resources and platforms.^43^ Addressing this gap requires modeling approaches that harmonize and integrate heterogeneous datasets and extract mechanistic information to improve model accuracy and biological interpretability.

This study presents a hierarchical computational modeling framework that integrates concentration–response HTS data, curated biological pathway information, and machine learning-based modeling to predict chemical toxicity across multiple endpoints. Specifically, this framework (1) maps concentration–response HTS data to molecular targets to quantify protein interactions (protein scores); (2) aggregates protein scores to characterize pathway-level perturbations (pathway scores), which summarize the integrated biological response of each pathway in response to chemical exposure; and (3) associates pathway scores with toxicity outcomes, incorporating *in vivo* exposure estimates to refine predictive accuracy. This approach addresses challenges related to data sparsity, heterogeneity, and biological complexity by improving the model performance and enhancing mechanistic interpretability. Integrating concentration–response data with hierarchical pathway modeling provides a scalable and interpretable strategy for predictive toxicology and supports the development of NAMs for chemical risk assessment.

## Methods

### *In Vivo* Toxicity Data Collection

The *in vivo* toxicity data set for this study was compiled from prior in-house modeling studies^44–46^ and publicly available literature sources.^47–49^ This data set includes acute oral systemic toxicity (LD_50_), maternal and prenatal developmental toxicity (NOAEL), and binary classifications for human and preclinical hepatotoxicity. These five toxicity endpoints were selected from previously curated data sets informed by earlier modeling studies to maintain methodological continuity and support comparative and mechanistic evaluation. Chemical structures were standardized using International Chemical Identifiers (InChI) for cross-source consistency.^49^ After curation, the final *in vivo* toxicity data set for model training comprised 10,781 unique chemicals.

Acute systemic toxicity data were collected from oral rodent studies and aggregated from multiple databases, covering 8699 unique chemicals.^44,47,49^ For compounds with multiple LD_50_ records, the lowest dose (i.e., the most potent result) was retained to conservatively estimate toxicity.

Maternal and prenatal developmental toxicity data were derived from oral rodent studies, comprising 446 unique compounds with developmental toxicity results and 375 unique compounds with maternal NOAEL data.^46^ All 375 compounds assessed for maternal toxicity were also present in the developmental data set. Evaluated outcomes included malformations, embryonic lethality, growth retardation, and *in utero* functional impairments.

Hepatotoxicity data were obtained from Mulliner et al.,^48^ which included experimental *in vivo* outcomes from preclinical animal studies and clinical observations in humans. The complete hepatotoxicity data set contained 3250 unique compounds with preclinical outcomes and 2171 unique compounds for human hepatotoxicity records, of which 1709 compounds overlapped between the two sets. Human data were derived from clinical trials, postmarket adverse event reports, and curated literature sources. The preclinical subset included pharmaceutical and industrial compounds, although some lacked detailed animal toxicity documentation.^48^

Acute, maternal, and developmental toxicity were modeled as continuous endpoints (i.e., LD_50_ and NOAEL values) to quantify variations in toxic potency among compounds. In contrast, hepatotoxicity was treated as a binary classification based on reported adverse liver outcomes, reflecting the categorical nature of available *in vivo* data and supporting toxicant classification within the same modeling framework.

### HTS Data Preprocessing and Standardization

HTS data were obtained from PubChem (https://pubchem.ncbi.nlm.nih.gov/, accessed December 1, 2024) and mapped to human-specific pathways using predefined gene–pathway associations in WikiPathways (https://www.wikipathways.org/, accessed December 1, 2024). WikiPathways provides an open, community-curated collection of human pathways, featuring standardized pathway representations and programmatic interfaces that enable quantitative integration with gene-level HTS data within the scope of this study.^50^ Assays were curated to retain only those with valid identifiers (PubChem Assay ID [AID], Compound ID [CID], and Substance ID [SID]) and available concentration–response data parametrized by the Hill equation.

For each compound–assay pair, Hill model parameters were extracted: AC_50_ (concentration at half-maximal effect), Hill slope (curve steepness), and Top (maximum modeled response). The Hill model defines the fitted concentration–response *R*(C) at concentration *C* as

$$R(C)=\mathrm{Top}\times \frac{C^{\mathrm{slope}}}{C^{\mathrm{slope}}+{\mathrm{AC}}_{50}^{\mathrm{slope}}}$$ 1

To characterize compound-induced response behavior, the Top parameter was extracted as a summary statistic representing the asymptotic response level predicted by the Hill model at high concentrations. Positive Top values indicate activation, whereas negative values indicate inhibition. For each assay, the minimum and maximum Top values observed in tested compounds were computed and denoted as minTop and maxTop, respectively. Assays were classified into three categories based on the direction and range of the modeled Top values, reflecting distinct underlying mechanisms of action: positive (minTop > 0 and maxTop > 0), negative (minTop < 0 and maxTop < 0), and mixed (minTop < 0 and maxTop > 0). Assays not meeting these criteria, including those with near-zero Top values (minimal modeled activity), contradictory response trends (e.g., minTop > 0 and maxTop < 0), or curves remaining near baseline, were excluded. These assays, exhibiting insufficient or inconsistent response variation among compounds, were considered uninformative for mechanistic interpretation or downstream modeling due to limited utility.

Concentration–response data for all compound–assay pairs were evaluated on a standardized grid of 45 logarithmically spaced concentrations ranging from 1 pM to 100 μM to ensure uniform input dimensionality for all assays.^30^ Response values were computed at these concentrations for each pair using the fitted Hill equation parameters (AC_50_, Hill slope, and Top). Modeled responses were normalized by rescaling the fitted baseline to 0% and the compound-specific Top value to 100%, yielding a bounded activity scale from 0 to 100%. This transformation preserved the direction and shape of the fitted response curve while enabling cross-assay and cross-compound comparisons (harmonization). When multiple concentration–response data sets for the same chemical-assay pair were available from different sources, readouts were first normalized to the logarithmic concentration range. Responses were then scaled to a standardized interval between 0 and 100%. Overlapping concentration ranges were interpolated, and quality control filters were applied to retain only the most reproducible profiles. The harmonized data sets were assembled into a three-dimensional tensor of shape (*n*_assays_ × *n*_concentrations_ × *n*_chemicals_), referred to as *Assay*, and used as the input for subsequent protein score modeling.

### Hierarchical Modeling, Statistical Filtering, and Model Training

A hierarchical modeling framework was implemented to represent chemical-induced bioactivity and predict toxicity scores using normalized HTS concentration–response data. The model architecture comprised three sequential layers, each represented by a matrix operation: *M*_1_ (assay-to-protein), *M*_2_ (protein-to-pathway), and *M*_3_ (pathway-to-toxicity). Each layer used matrix multiplication (tensor contraction) and gradient-based optimization implemented in PyTorch (v2.2.2+cu118, https://pytorch.org/).^51–53^

The input tensor, *Assay,* had dimensions (*n*_assays_ × *n*_concentrations_ × *n*_chemicals_), where each element represented a normalized Hill-fitted response for a given compound–assay pair. In the first transformation, a latent protein tensor *Prot* (*n*_proteins_ × *n*_concentrations_ × *n*_chemicals_) was introduced as a trainable parameter, and *Assay* was reconstructed from *Prot* through a learnable matrix *M*_1_ (*n*_assays_ × *n*_proteins_):

$$Assay=M_{1}\cdot Prot$$ 2

In the second layer, pathway activity (*Path*) was reconstructed from *Prot* using a learnable matrix *M*_2_ (*n*_proteins_ × *n*_pathways_):

$$Prot=M_{2}\cdot Path$$ 3

The resulting *Path* tensor (*n*_pathways_ × *n*_concentrations_ × *n*_chemicals_) encoded modeled bioactivity at a pathway level. This layer enabled the interpretation of activity as coordinated pathway perturbations, thereby improving mechanistic resolution compared to isolated protein responses. Matrices *M*_1_ and *M*_2_ were learned via backpropagation by minimizing the mean square error (MSE) between the reconstructed and observed input tensors.^54^ Training was performed using the Adam optimizer (initial learning rate = 0.001) and ReduceLROnPlateau learning rate scheduler (10% rate decay) triggered upon training loss plateauing.^55^ Training was conducted for up to 1000 epochs, with early stopping implemented upon convergence. Formal grid search or *k*-fold cross-validation was not applied because the modeling objective focused on hierarchical tensor reconstruction (i.e., mapping assay data to protein targets, pathways, and toxicity endpoints) rather than predictive generalization. Model robustness was assessed through repeated training with random initializations, which produced consistent convergence behavior and comparable tensor structures across iterations.

The final transformation introduced a trainable toxicity tensor (*Tox*) with shape (*n*_toxicities_ × *n*_concentrations_ × *n*_chemicals_). A supervised filtering matrix *M*_3_ (*n*_pathways_ × *n*_toxicities_) was applied to *Tox* to reconstruct the modeled pathway scores (*Path*):

$$Path=M_{3}\cdot Tox$$ 4

*Tox* was optimized so that the transformed product in eq 4 approximated the modeled *Path* tensor. Unlike *M*_1_ and *M*_2_, which were learned in an unsupervised manner, *M*_3_ was derived using supervised statistical filtering to retain biologically plausible and statistically significant (*q* < 0.05) pathway–endpoint associations.

For continuous toxicity endpoints (−log-transformed LD_50_ for acute systemic toxicity and −log-transformed NOAEL for maternal and prenatal developmental toxicities), Pearson correlation coefficients were computed between *Path* scores and observed toxicity endpoint values. A pathway-endpoint pair was retained in *M*_3_ only if it met all of the following criteria: (1) Pearson correlation coefficient *r* ≥ 0.1; (2) false discovery rate (FDR)-adjusted *q* < 0.05 using the Benjamini-Hochberg method;^56^ and (3) a sample size-dependent threshold, $|r|=\frac{2}{\sqrt{n}}$ , where *n* is the number of compounds associated with that pathway–endpoint pair.^57–60^

The *r* ≥ 0.1 cutoff was chosen to capture moderate but biologically meaningful associations that can occur in high-dimensional data, while FDR correction controlled for multiple testing. Sensitivity analysis across correlation thresholds (0.05 ≤ *r* ≤ 0.2) yielded comparable sets of pathway–endpoint associations, indicating that the model results were robust to reasonable threshold variations. The Fisher *z*-transformation was applied to estimate 95% confidence intervals (CIs) for *r* assuming approximate normality.^61^ For LD_50_ data, additional CIs were obtained through nonparametric bootstrapping to better represent variability in acute systemic toxicity measurements.^62,63^

For binary endpoints (human and preclinical hepatotoxicity), nonparametric comparisons of *Path* score distributions between toxic and nontoxic groups were performed. The Wilcoxon rank-sum test was used in the absence of ties; otherwise, the Brunner–Munzel test was applied (i.e., identical scores that could not be differentiated). Effect size was expressed as the rank-biserial correlation (*r*_rb_), with 95% CIs estimated using Fisher *z*-transformation.^61^ A binary pathway-endpoint pair was retained in *M*_3_ if *r*_rb_ > 0 and FDR-adjusted *q* value < 0.05.

Following filtering, *M*_3_ was binarized to define the final supervised transformation structure. The resulting *Tox* tensor was reoptimized by minimizing MSE between reconstructed *Path* scores (eq 4) and upstream modeled *Path* tensors. This layer followed the same training protocol as earlier layers *M*_1_ and *M*_2_ (i.e., Adam optimizer with the learning rate scheduler and early stopping). The supervised training of *M*_3_, which relied on the filtering process, distinguished this layer from the unsupervised ones. After training, *Tox* scores were normalized within each endpoint group using min–max scaling to [0, 1] for cross-chemical comparability.

All mappings (i.e., assay-to-protein, protein-to-pathway, and pathway-to-toxicity) used unweighted MSE. To mitigate the disproportionate influence of endpoints with larger sample sizes without introducing potential biases, the pathway-to-toxicity mapping was restricted to pathway–endpoint pairs meeting a significance threshold (*p* < 0.05) and minimum correlation thresholds. After training, toxicity scores were normalized within each endpoint. Sample sizes for each endpoint were reported alongside the results to support the interpretation of model performance.

### Toxicokinetic Modeling for Exposure Integration

To examine toxicity predictions within realistic exposure conditions, toxicokinetic (TK) modeling was employed to estimate steady-state plasma concentrations (*C*_ss_) for a subset of compounds. *C*_ss_ values were computed using the EPA’s *httk* package (https://cran.r-project.org/web/packages/httk/index.html, accessed December 1, 2024), which implements physiologically based pharmacokinetic modeling to simulate internal plasma (i.e., steady-state) concentrations under specified external dose scenarios.^64^ Estimates were generated assuming a standardized repeated-dose oral exposure of 1 mg/kg/day.

*C*_ss_ values were available for 567 compounds with corresponding HTS assay data and TK parameters provided in the *httk* package. Compounds used solely for curve normalization or model calibration were excluded. *C*_ss_ values were independently calculated and applied only during post hoc evaluation under a standardized 1 mg/kg/day oral dosing scenario to ensure cross-compound comparability. These exposure integration results are constrained by the availability of toxicokinetic data coverage and the assumptions inherent in the dosing scenario. Although this standardized approach enables consistent evaluation, the modeling framework remains flexible and can incorporate compound-specific or population-based exposure estimates in future applications.

To represent interindividual variability in clearance and metabolism, *C*_ss_ was modeled as a population distribution. The fifth percentile represented individuals with higher metabolic capacity and clearance (i.e., lower *C*_ss_), while the 95th percentile represented individuals with slower clearance (i.e., higher *C*_ss_), approximating potential responses in sensitive subpopulations. The 95th percentile *C*_ss_ for each compound was used in case study evaluations to compare modeled exposure estimates against predicted bioactivity at the *Assay*, *Prot*, and *Path* levels. These comparisons assessed whether modeled effects occurred at concentrations relevant to internal human exposure, thereby supporting hazard interpretation within the NAMs framework.^29^

## Results and Discussion

### Hierarchical Modeling Workflow for Predicting Chemical Toxicity

Figure 1 illustrates the hierarchical modeling approach developed to integrate concentration–response HTS assays from PubChem with filtered biological pathway definitions from WikiPathways for toxicity prediction. This workflow translates*in vitro* bioactivity into structured biological scores at multiple mechanistic levels, generating interpretable toxicity predictions for complex endpoints.

**Figure 1:**
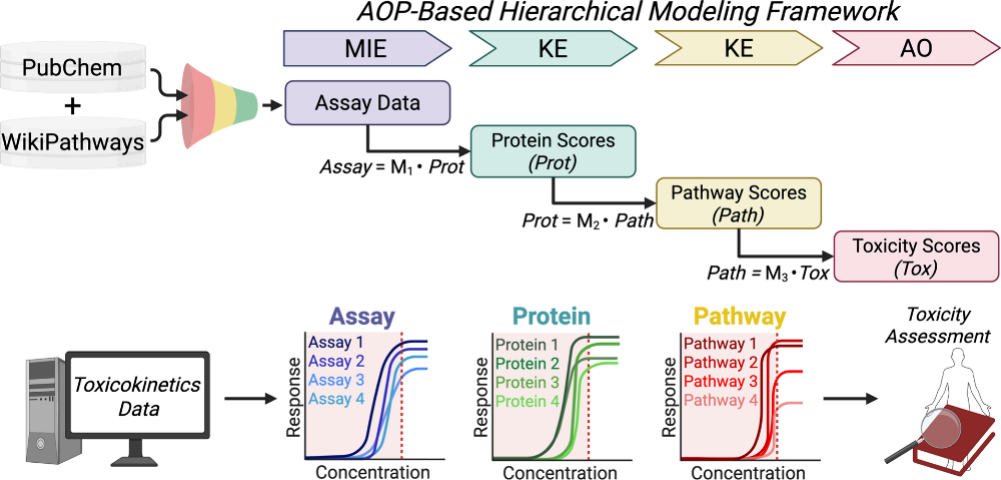
Adverse outcome pathway (AOP)-based hierarchical concentration–response modeling framework for mechanistic chemical toxicity predictions. The workflow integrates high-throughput screening (HTS) concentration–response data from PubChem with curated biological pathway information from WikiPathways to model compound-level toxicity scores (*Tox*) using a multilayered approach. *Assay* data were first translated into protein scores (*Prot*) via transformation matrix *M*_1_ representing molecular initiating events (MIEs) and key events (KEs) (eq 2). *Prot* scores were aggregated into pathway scores (*Path*) via matrix *M*_2_, summarizing higher-order biological responses through mechanistically related targets (i.e., combinations of related MIEs and KEs) (eq 3). *Path* scores were reconstructed from a trainable toxicity tensor (*Tox*) using matrix *M*_3_, a fixed matrix representing filtered pathway–toxicity associations (eq 4). Modeled concentration–response behavior is shown for representative *Assay*, *Prot*, and *Path* scores. Toxicokinetic steady-state concentrations (C_ss_) were estimated independently to improve model predictivity for exposure-informed chemical toxicity assessment. Created with Biorender.com.

First, normalized concentration–response profiles from HTS assays were compiled into a three-dimensional *Assay* tensor, representing compound responses across assays and concentrations. These data served as input to the first layer of the model, where HTS assay responses were mapped to *Prot* scores using matrix *M*_1_. The resulting *Prot* tensor encodes modeled chemical-protein interactions, corresponding to MIEs and KEs (eq 2).

Second, *Prot* scores were aggregated into *Path* scores through transformation by matrix *M*_2_ (eq 3). This step summarizes bioactivity at the pathway level, reflecting perturbations of mechanistically related proteins. The *Path* tensor was subsequently used to generate *Tox* scores through matrix *M*_3_, which was constructed using a supervised learning process of pathway–endpoint relationships (eq 4). The resulting *Tox* scores represent modeled AOs across five endpoints: acute systemic toxicity, maternal and developmental toxicity, and human and preclinical hepatotoxicity.

Third, *C*_ss_ values were estimated using TK modeling for chemicals with available parameters. These estimates were applied to identify compounds for which modeled *in vivo* exposures could reach or exceed predicted effect concentrations across the *Assay*, *Prot*, and *Path* levels. *C*_ss_ was incorporated post hoc to evaluate the exposure relevance of modeled predictions.

Finally, outputs from the hierarchical model were analyzed to identify biological features (i.e., assays, proteins, pathways) contributing to predicted toxicity. Assay-level responses (stored in the *Assay* tensor), along with *Prot* scores, and *Path* scores, were examined to identify the key biological drivers of the final *Tox* scores. These analyses support the mechanistic interpretation of chemical effects and enable downstream applications, such as compound grouping, pathway-informed hazard evaluation, and data structuring for model transparency and regulatory decision support.

### Assay Selection and Protein Mapping

From an initial set of 297,803 PubChem assays targeting genes mapped to WikiPathways, a total of 1247 assays were identified that contained valid concentration–response data and could be parametrized using the Hill equation (eq 1). These assays corresponded to 371 unique genes which were retained for integration into the modeling framework. Hill model parameters were extracted for each compound–assay pair (eq 1), and assay-level responses were characterized by the Top parameter, which estimates the asymptotic maximum (or minimum) response achieved at high concentrations.

To enable comparability between assays and compounds, we normalized concentration–response curves using assay-specific minimum and maximum Top values, denoted as minTop and maxTop, respectively. These values defined the lower and upper plateaus of fitted response curves observed across all tested compounds within each assay. This normalization using these boundaries demonstrated the overall range of functional activity, preserved the biological relevance of the assay mechanism, minimized noise from inactive compounds, and enabled standardized comparison of compound activity across assays (Figure 2A).

**Figure 2:**
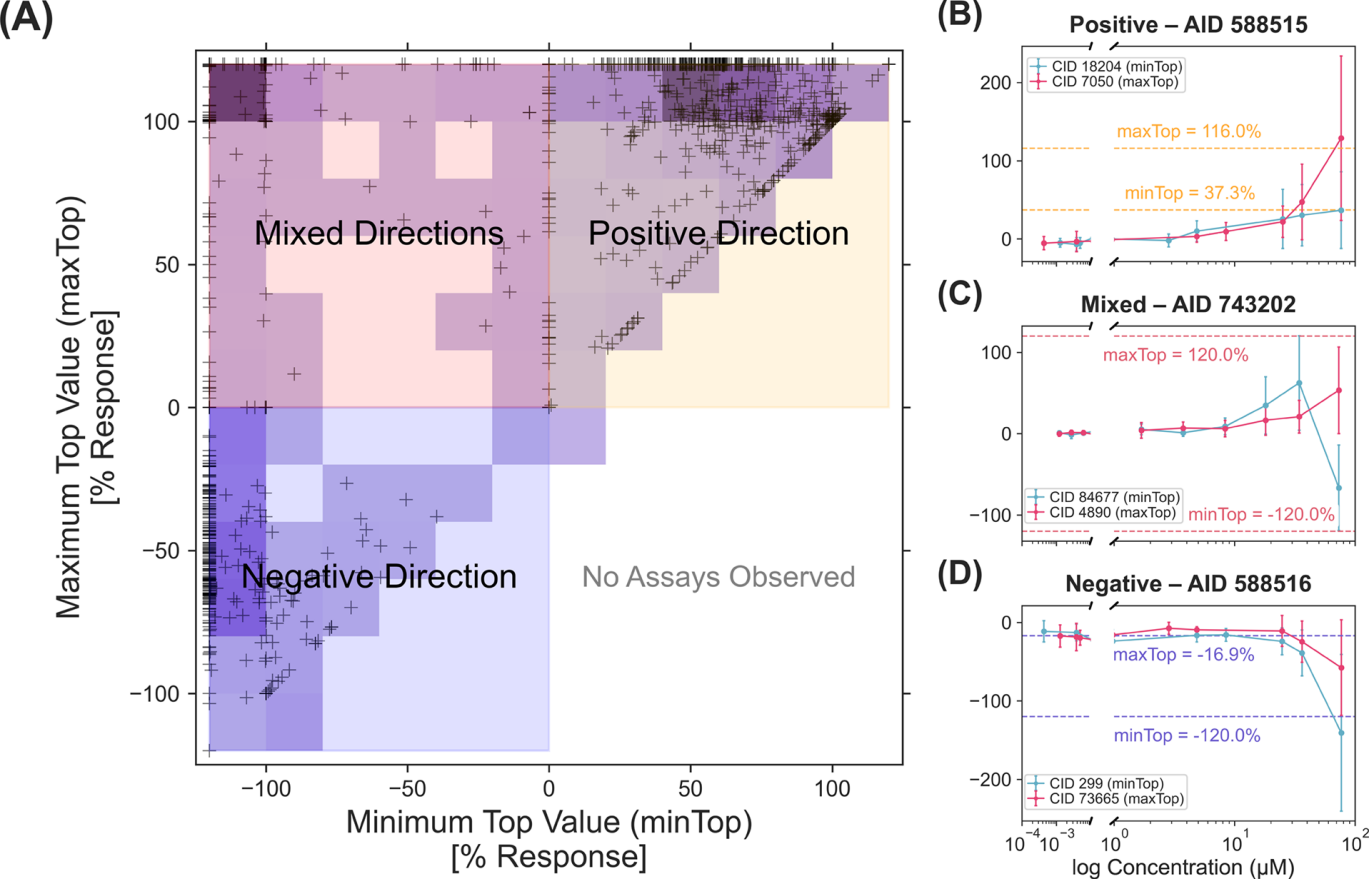
Classifying assays by mechanistic activity patterns from concentration–response behavior. (A) Each point represents an HTS assay positioned by its minimum (minTop, *x*-axis) and maximum (maxTop, *y*-axis) fitted response values across all compounds tested in that assay. Assays were classified into quadrants according to directional response patterns: positive (top-right; minTop > 0, maxTop > 0), mixed (top-left; minTop < 0, maxTop > 0), negative (bottom-left; minTop < 0, maxTop < 0), and undefined (bottom-right; minTop > 0, maxTop < 0), for which no assays were observed. Shading colors show assay density, with darker regions indicating a greater number of classified assays. (B–D) Representative assays for each classification category are shown with fitted concentration–response curves for the compounds corresponding to the minTop and maxTop values.

Among the 1247 curated assays, 641 exhibited positive minTop and maxTop values, indicative of agonist-like behavior in which most compounds induced upward signal shifts following measurable activation of the assay target. An example of a positive Top assay was the quantitative HTS (qHTS) assay for small molecule agonists of androgen receptor signaling (AID 588515; minTop = 37.3%, maxTop = 116%) (Figure 2B). In contrast, 304 assays displayed negative minTop but positive maxTop values, indicating mixed mechanisms (i.e., heterogeneous response) across testing compounds, such as partial agonism and inverse agonism. An example assay with this mixed response profile was the qHTS assay for small molecule agonists of the antioxidant response element (ARE) signaling pathway (AID 743202; minTop = −120%, maxTop = 120%) (Figure 2C). The remaining 302 assays exhibited negative minTop and negative maxTop values, emulating an antagonist-like behavior of the tested compounds. A representative assay of this group was the qHTS assay for small molecule antagonists of androgen receptor signaling (AID 588516; minTop = −16.9%, maxTop = −120%) (Figure 2D).

By definition, the Hill model constrains the maximum to exceed the minimum; thus, assays in the bottom right quadrant (positive minTop and negative maxTop) were not theoretically valid (Figure 2A). This quadrant-based classification revealed groupings of assays by directionality in compound responses, despite variability in curve shape, noise, or resolution across individual concentration–response profiles (Figure 2B–D).

For modeling purposes, the initial assay data set was filtered to include only assays with valid Hill model parameters and compound–assay pairs that were present in the toxicity data set and exhibited measurable activity suitable for model training. Of the 1247 identified assays with concentration–response data, not all assays had adequate parametrization for modeling due to missing or invalid Hill fits. After filtering, 455 assays fulfilled the inclusion criteria and were retained for integration into the protein-layer modeling step (eq 2). Among the retained assays, the majority (431 of 455 assays) tested compounds at five or more unique concentrations, with an average of 11 concentrations per compound across the data set. The number of concentrations tested per compound ranged from two concentrations, as observed in a qHTS assay targeting inhibitors of the extracellular signal-regulated kinase (ERK) signaling pathway (AID 1454), to 46 concentrations, as reported in a qHTS assay for small molecule agonists of the p53 signaling pathway (AID 651631).

The filtered assay set was mapped to 216 unique protein targets using assay–protein relationships provided in PubChem. The complete list of protein targets and their mapped assays is provided in Supplemental Table 1, with a subset of proteins having five or more assays is shown in Table 1. On average, each protein was associated with two assays. This mapping process was used to compute *Prot* scores, which quantified compound-specific interactions with 216 protein targets using corresponding assay-level responses.

**Table 1:** Genes with Five or More Associated HTS Assays from PubChem Containing Concentration–Response Data

**Wikipathways gene symbol**	**Protein name**	**Gene ID**	**Number of **assays
NFE2L2	NFE2 like bZIP transcription factor 2	4780	11
PKM	pyruvate kinase M1/2	5315	10
VDR	vitamin D receptor	7421	9
ESR1	estrogen receptor 1	2099	9
MITF	melanocyte inducing transcription factor	4286	9
THRB	thyroid hormone receptor beta	7068	8
AR	androgen receptor	367	8
ESRRA	estrogen-related receptor alpha	2101	8
GMNN	geminin DNA replication inhibitor	51053	8
HTT	huntingtin	3064	7
RAB9A	RAB9A, member RAS oncogene family	9367	7
ABHD5	abhydrolase domain containing 5, lysophosphatidic acid acyltransferase	51099	7
MTOR	mechanistic target of rapamycin kinase	2475	6
PPARG	peroxisome proliferator activated receptor gamma	5468	5
CASP3	caspase 3	836	5
NPC1	NPC intracellular cholesterol transporter 1	4864	5
CYP3A4	cytochrome P450 family 3 subfamily A member 4	1576	5
KMT2A	lysine methyltransferase 2A	4297	5
NFKB1	nuclear factor kappa B subunit 1	4790	5
TP53	tumor protein p53	7157	5
AICDA	activation-induced cytidine deaminase	57379	5

### Chemical–Protein Response Analysis

The nuclear factor erythroid 2-like 2 (NFE2L2, commonly referred to as Nrf2), is a transcription factor that regulates antioxidant and cytoprotective gene expression in response to oxidative stress.^65,66^ This protein target was mapped to 11 HTS assays retained after filtering for valid concentration–response data (Table 1). *Prot* scores for Nrf2 were computed for 470 compounds using matrix *M*_1_ (eq 2), which mapped assay-level responses (*Assay*) to protein-level activity. *Prot* scores for Nrf2 ranged from −1.5 to 1, representing modeled compound-specific modulation of Nrf2 activity derived from integrated HTS responses.

The resulting *Prot* values distinguished two major classes of modeled mechanisms of action: repression and activation of Nrf2 signaling. This differentiation paralleled the bidirectional activity trends observed in the ARE signaling assay (Figure 2C), with negative and positive fitted responses corresponding to compounds that modulate this stress-response pathway. Compounds with negative Nrf2 *Prot* scores, including the glucocorticoid analogs (Figure 3), shared steroid-like structural features and were associated with repression of Nrf2 signaling.^67,68^ Glucocorticoids reduce oxidative stress and proinflammatory signaling, thereby diminishing the cellular demand for Nrf2-mediated antioxidant responses.^69^ Conversely, compounds with positive *Prot* scores were modeled to activate Nrf2. Two examples, 2-chloro-5-nitro-N-phenylbenzamide (CAS 22978-25-2, *Prot* = 1) and pifexole (CAS 27199-40-2, *Prot* = 0.5), contain electrophilic substituents within a benzamide scaffold that may promote dissociation of Nrf2 from its negative regulator Keap1, allowing Nrf2 to translocate into the nucleus, and then activate the transcription of antioxidative genes.^70–72^ The findings demonstrate that *Prot* scores derived from assay-level responses can infer compound-level directionality of Nrf2 modulation and identify structure–activity relationships with known mechanisms of action.

**Figure 3:**
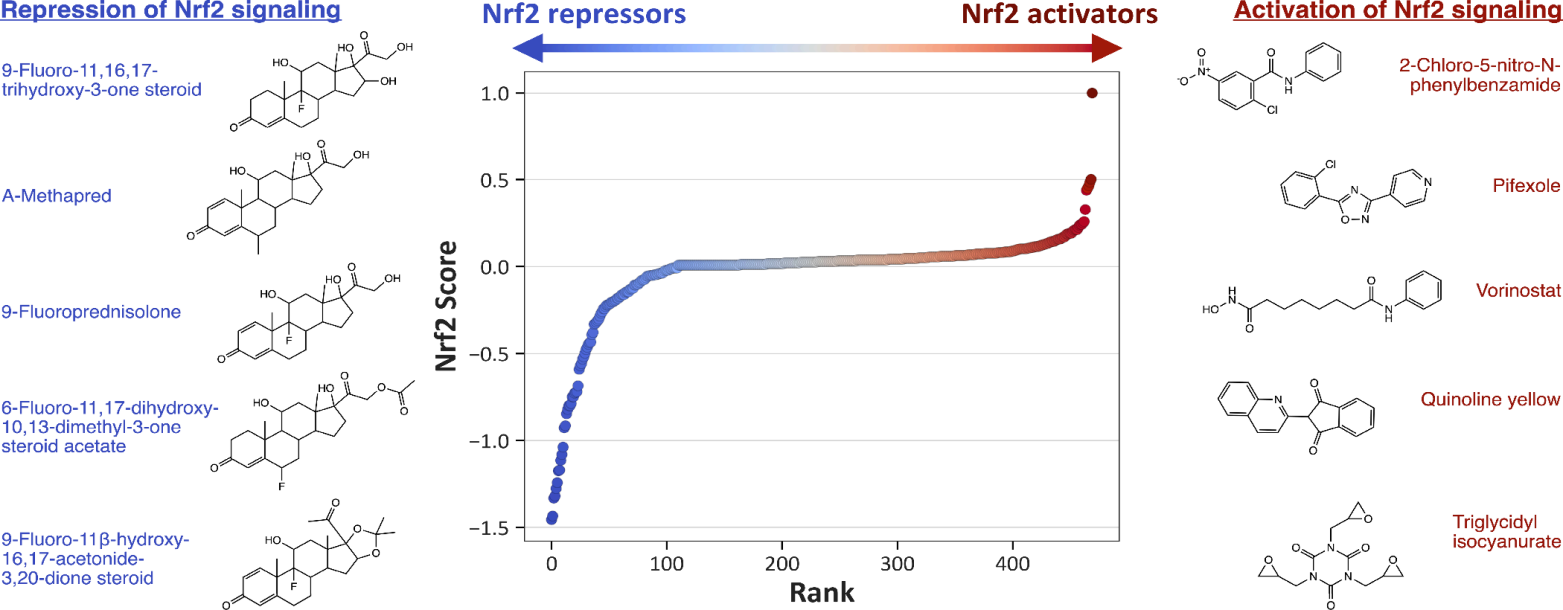
Compounds (individual dots) ranked by modeled Nrf2 protein scores (*Prot*), with the top five repressors (left, in blue) and activators (right, in red) shown alongside associated chemical structures and names.

### Chemical Clustering and Toxicity Profiles

To examine structure–activity relationships between chemical structure, protein-level activity, and *in vivo* toxicity outcomes, compounds were clustered using MACCS fingerprints and the Taylor–Butina algorithm with a Tanimoto similarity threshold of 0.7, grouping compounds by shared substructural similarity.^73^ Clusters with fewer than five compounds were excluded, resulting in 33 clusters retained for analysis (Figure 4A). The largest shared substructure for each cluster was encoded as a SMARTS pattern. Continuous toxicity endpoints (i.e., acute systemic toxicity [LD_50_] and developmental toxicity [NOAEL]) were log-transformed using a negative base-10 logarithm, following the approach of previous studies.^37,44^ Cluster-level *z*-scores were computed by standardizing the median toxicity value for each cluster against the mean and standard deviation of all compound-level values for that endpoint. For binary endpoints (i.e., human and preclinical hepatotoxicity), binary values were classified as 0 or 1, converted to z-scores, and multiplied by −1 to preserve directional consistency with continuous toxicity endpoint values. On this scale, higher *z*-scores indicate increased toxicity across all endpoints. Cluster toxicity profiles were compared using *in vivo* toxicity *z*-scores and modeled *Prot* scores.

**Figure 4:**
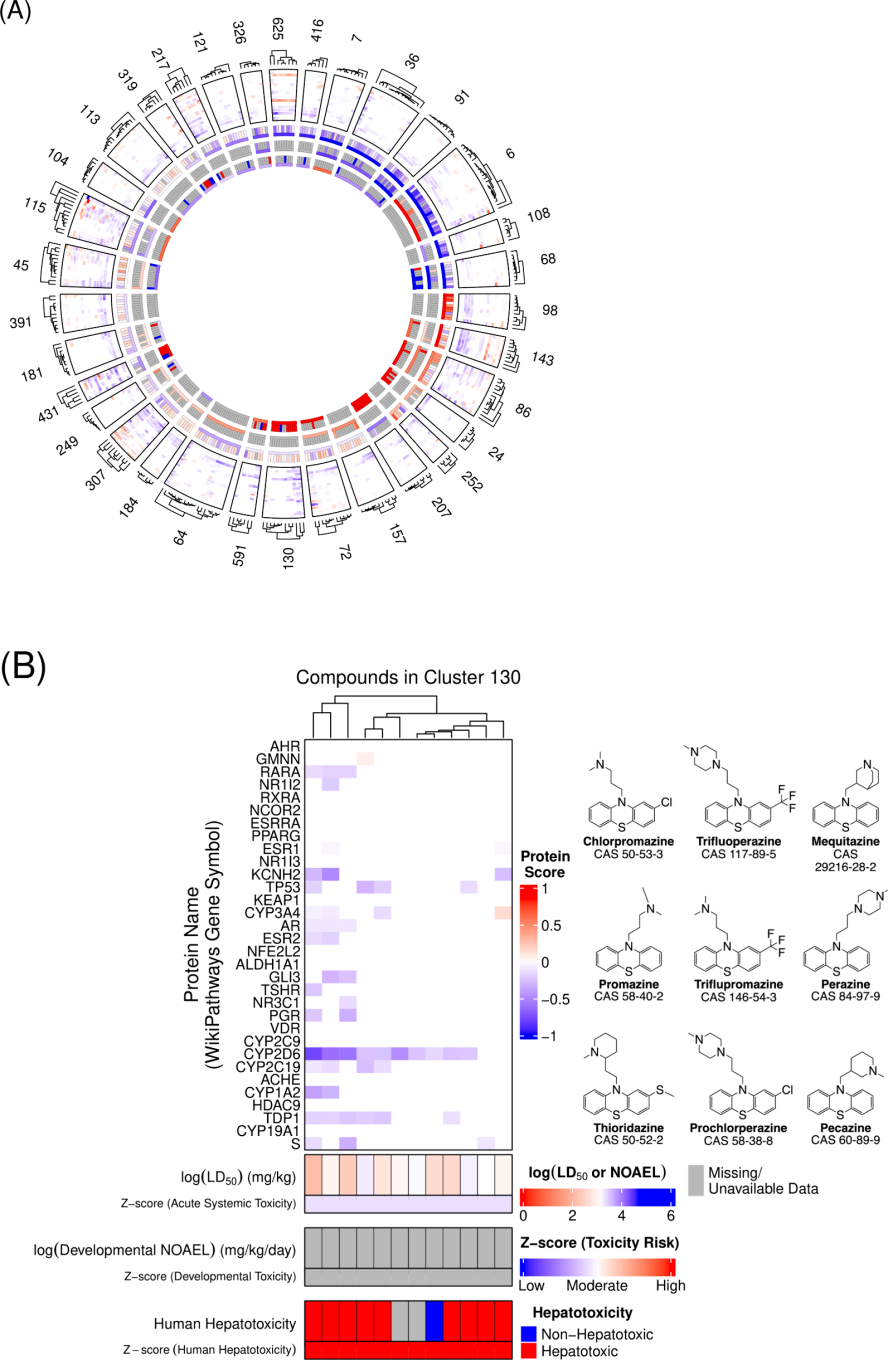
Structural clusters of potential toxicants with associated protein scores (*Prot*) and *in vivo* toxicity profiles. (A) Each sector represents one of 33 structural clusters (≥ five compounds), defined using MACCS fingerprints and the Taylor–Butina algorithm (Tanimoto similarity ≥ 0.7) and sorted by the mean toxicity *z*-scores across toxicity endpoints. The outer heatmap displays modeled *Prot* scores (rows: proteins; columns: compounds), with red and blue indicating positive and negative perturbations, respectively; white indicates scores near zero, representing no modeled activity. The outer hierarchical dendrogram depicts compound similarity relationships within clusters. The three innermost concentric rings show measured *in vivo* toxicity endpoints: acute oral systemic toxicity (LD_50_, top ring), developmental toxicity (NOAEL, middle ring), and human hepatotoxicity (binary, bottom ring). Log-transformed continuous toxicity values and binary hepatotoxicity are shown (top row) with the corresponding *z*-scores (bottom row). Colors range from red (higher *z*-score values/higher relative toxicity) to blue (low *z*-score values/low relative toxicity); gray indicates missing values. (B) Cluster 130, enlarged from (A), includes nine compounds classified as human hepatotoxicants.

Distinctive toxicity profiles were observed across clusters. Cluster 130, composed of phenothiazines, was associated with human hepatotoxicity (*z*-score = 1.2) but had low acute systemic toxicity (*z*-score = −0.3) (Figure 4B, Supplemental Table 1). Phenothiazines are neuroleptic agents with known hepatotoxic risk, often attributed to CYP-mediated metabolic activation and formation of reactive intermediates that contribute to drug-induced liver injury (DILI).^74,75^ Most Cluster 130 compounds had negative *Prot* scores for CYP3A4, CYP2D6, and CYP2C19, indicating inhibitory activity that may impair metabolic clearance and increase hepatic stress. These hepatotoxic compounds also showed negative *Prot* scores for the SARS-CoV-2-surface glycoprotein (S), a viral target included in the model.^76^ One exception, pecazine (CAS 60-89-9), showed a positive CYP3A4 *Prot* score (0.14) and was previously classified at the highest DILI concern level.^77,78^ Although direct evidence linking pecazine to CYP-mediated hepatotoxicity remains limited, the observed interaction may suggest alternative mechanisms of hepatic injury that are not fully elucidated by current profiles.

Cluster 6 ranked highest for developmental toxicity among all clusters (*z*-score = 3.7; median NOAEL of 64 mg/kg bw/day), indicating adverse effects at relatively low exposure levels.^79,80^ Among the 21 compounds, 11 showed negative CYP1A2 *Prot* scores (mean = −0.16), and 14 compounds showed negative CYP2C19 *Prot* scores (mean *Prot* = −0.11), excluding inactive scores (*Prot* = 0). These data suggest limited modeled interaction with these CYP enzymes, implying that metabolic activation through CYP1A2 or CYP2C19 is not required for the observed toxicity in this cluster. In contrast, acute systemic toxicity was minimal, with high LD_50_ values (LD_50_ *z*-score = −10.9), indicating that these compounds were not generally toxic under acute exposure conditions. The combination of low developmental NOAELs and low acute toxicity supports a mechanism involving pathway-specific and direct developmental disruption at sublethal doses rather than generalized acute systemic toxicity.

Cluster 98, composed primarily of organophosphate compounds, exhibited the highest acute systemic toxicity among all clusters (*z*-score = 7.9), with a low median LD_50_ of 10 mg/kg. These compounds are widely used as pesticides and share a well-established mechanism of action for acute toxicity involving irreversible inhibition of acetylcholinesterase (AChE), leading to cholinergic crisis and respiratory failure.^81,82^ Modeled protein-level interactions showed modest CYP3A4 inhibition (*Prot* range: −0.1 to −0.5), indicating secondary metabolic involvement but not a primary role in the AChE-driven mechanism.^83^

Cluster 207, composed entirely of sulfonamides, exhibited the highest hepatotoxicity (*z*-score = 3.5) among all clusters. Sulfonamide-induced liver injury is typically idiosyncratic DILI, with mechanisms attributed to metabolic bioactivation and immune-mediated responses.^84,85^ Several Cluster 207 compounds showed negative *Prot* scores for CYP enzymes, including CYP3A4 (cyclothiazide, CAS 2259-96-3: −0.23; althiazide, CAS 5588-16-9: −0.24), CYP2C9 (penflutizide, CAS 1766-91-2: −0.34, and CYP2D6 (penflutizide: −0.27). These reductions in modeled CYP enzyme activity may impair detoxification and promote metabolite accumulation. Together, these results highlight how integrating structural clustering with protein-level activity modeling delineates chemical groups with mechanistic associations to toxicity outcomes.

### Pathway Mapping and Identification of Associated Pathways with Toxicity Outcomes

A total of 216 proteins from the hierarchical model were mapped to 550 biological pathways obtained from WikiPathways. For each compound, *Path* scores were computed using matrix transformation *M*_2_, aggregating *Prot* scores into pathway-level perturbation profiles across tested concentration ranges (eq 3). Because not all 550 pathways were expected to contribute to toxicity outcomes, statistical filtering was applied to identify biologically and statistically relevant associations. Pathways were retained if their *Path* scores showed a statistically significant and positive correlation with toxicity outcomes (*r* ≥ 0.1 for continuous endpoints, *r*_*rb*_ > 0 for binary endpoints; *q* < 0.05). This supervised feature selection process ensured that only pathways with increasing toxicity potential were included in the downstream supervised modeling stage. Through this filtering process, 103 unique pathways were identified as statistically associated with at least one toxicity endpoint: 24 pathways correlated with acute systemic toxicity, 24 with maternal toxicity, 31 with developmental toxicity, 7 with human hepatotoxicity, and 41 with preclinical hepatotoxicity (Supplemental Table 2). Overlap among 20 pathways associated with more than one toxicity endpoint revealed shared biological mechanisms underlying diverse toxicity outcomes.

Table 2 lists the top five significant pathways for each endpoint, ranked by correlation strength. The statistical relationships between pathway perturbations and toxicity outcomes are illustrated in Supplemental Figure 1. For continuous endpoints, linear regression analyses between *Path* scores and log-transformed toxicity values demonstrated positive correlations (Supplemental Figure 1A). For binary hepatotoxicity, boxplots compared *Path* score distributions between hepatotoxic and nonhepatotoxic compounds, with corresponding correlation statistics (Supplemental Figure 1B). These analyses confirmed that selected pathway perturbations (i.e., *Path* scores) were statistically correlated with toxicity endpoints and reflected plausible mechanisms supported by prior literature.

**Table 2:** Top Five Statistically Significant Pathways for Each Toxicity Endpoint, Ranked by Correlation Strengths (*r* or *r*_rb_) from Pathway–Toxicity Correlations

Pathway (WikiPathways ID)	Correlation	*q* value	Toxicity endpoint
WP3851: TLR4 signaling	0.197	2.75 × 10^–17^	acute
WP2884: NRF2	0.192	2.03 × 10^–16^	acute
WP5095: proinflammatory and profibrotic mediators	0.141	2.50 × 10^–08^	acute
WP5050: TCA cycle in senescence	0.139	2.66 × 10^–08^	acute
WP2491: diclofenac metabolism	0.13	2.97 × 10^–07^	acute
WP4545: oxysterol	0.303	0.00135	maternal
WP1992: miRNA and adipocytes	0.271	0.00511	maternal
WP4659: gastrin signaling	0.27	0.00516	maternal
WP2064: neural crest differentiation	0.261	0.00726	maternal
WP3915: angiopoietin-like protein 8 regulatory	0.253	0.0104	maternal
WP5117: cohesin complex—Cornelia de Lange syndrome	0.313	0.000281	developmental
WP4880: host–pathogen interaction of human coronaviruses—interferon induction	0.302	0.000534	developmental
WP3915: angiopoietin-like protein 8 regulatory	0.286	0.00117	developmental
WP4725: sphingolipid metabolism	0.284	0.00125	developmental
WP4549: fragile X syndrome	0.282	0.00142	developmental
WP2873: aryl hydrocarbon receptor signaling	0.089	0.00144	human hepatotoxicity
WP4249: hedgehog signaling	0.069	0.00752	human hepatotoxicity
WP2012: miRNA in muscle cell differentiation	0.063	0.0253	human hepatotoxicity
WP2436: dopamine metabolism	0.063	0.0253	human hepatotoxicity
WP3298: melatonin metabolism	0.062	0.0367	human hepatotoxicity
WP2640: aripiprazole metabolism	0.137	2.32 × 10^–08^	preclinical hepatotoxicity
WP1604: codeine and morphine metabolism	0.135	2.66 × 10^–08^	preclinical hepatotoxicity
WP3893: development and heterogeneity of the ILC family	0.11	5.10 × 10^–06^	preclinical hepatotoxicity
WP2880: glucocorticoid receptor pathway	0.108	1.14 × 10^–05^	preclinical hepatotoxicity
WP5273: intestinal microbiome effect on vitamin K antagonists	0.1	4.80 × 10^–05^	preclinical hepatotoxicity

The model also identified novel pathway–toxicity relationships not previously characterized in *in vivo* studies, underscoring its potential to generate new mechanistic knowledge from large-scale HTS data. For example, TLR4 signaling (WP3851) has been implicated in acute renal and hepatic toxicity,^86,87^ the Cohesin Complex (WP5117) in developmental disorders such as Cornelia de Lange syndrome,^88^ and aryl hydrocarbon receptor signaling (WP2873) in hepatotoxic outcomes, including liver fibrosis and metabolic dysfunction-associated steatotic liver disease.^89,90^ These findings demonstrate that the hierarchical modeling approach successfully identified biologically meaningful pathway perturbations associated with specific toxicity outcomes.

In addition to confirming known pathways, this framework can identify undercharacterized or data-sparse pathways that lack mechanistic representation in public databases such as WikiPathways. Examples include Alström syndrome (WP5202), 2q13 copy number variation syndrome (WP5222), porto-sinusoidal vascular disease (WP5269), angiopoietin-like protein 8 regulatory (WP3915), UDP-derived sugars synthesis (WP5394), kynurenine and cell senescence (WP5044), and netrin–UNC5B signaling (WP4747) (Supplemental Table 2). Recognition of these pathways demonstrates the framework’s ability to prioritize mechanistically uncertain areas for targeted experimental investigation and to support refinement of NAMs. Moreover, the framework can inform development of new testing strategies for chemical classes with limited information on toxicity mechanisms through identification of novel assay–pathway relationships, contributing to the ongoing expansion and curation of pathway definitions in public resources (e.g., WikiPathways).

### Toxicity Scoring

*Tox* scores were computed for each compound by transforming *Path* scores through the learned *M*_3_ matrix (eq 4). We calculated scores for 3769 compounds across the five toxicity endpoints using the 103 statistically filtered pathways as input features. Each pathway contributed equally during model implementation.

Normalized *Tox* scores were used to rank compounds for each endpoint, and the highest-scoring compounds were identified accordingly. For example, cisapride (CAS 81098-60-4), a heartburn medication withdrawn due to severe cardiac side effects, had a normalized mean *Tox* score of 0.54. Compounds achieving the maximum normalized *Tox* score of 1 for each respective endpoint included niclosamide (CAS 50-65-7) for acute systemic toxicity, eliprodil (CAS 119431-25-3) for maternal toxicity, colforsin (CAS 66575-29-9) for developmental toxicity, NS-398 (CAS 123653-11-2) for human hepatotoxicity, and propiconazole (CAS 60207-90-1) for preclinical hepatotoxicity. These compounds were predicted to be highly toxic due to perturbation of the key pathways that most strongly contributed to the composite *Tox* scores.

Among these compounds, niclosamide, an oral anthelmintic approved for tapeworm infections, is classified as H302 (“harmful if swallowed”) and H400 (“very toxic to aquatic life”) under the European Union Classification, Labeling and Packaging (CLP) Regulation.^91^ Eliprodil, a noncompetitive *N*-methyl-d-aspartate (NMDA) receptor antagonist, was investigated in clinical trials for acute ischemic stroke but was discontinued because of insufficient efficacy.^92,93^ Direct maternal toxicity data on eliprodil were not identified; however, ketamine (CAS 6740-88-1), another NMDA receptor antagonist and structural analog, induces widespread neuroapoptosis in developing rat brain following maternal exposure, suggesting potential prenatal neurodevelopmental risk for this structural class.^94,95^ Colforsin (forskolin), a labdane diterpene that directly activates adenylyl cyclase, has been reported to disrupt cardiovascular development in chick embryos, although detailed mechanistic studies remain limited.^96^ NS-398, a selective COX-2 inhibitor structurally related to nimesulide, belongs to a class of compounds associated with idiosyncratic hepatotoxicity in humans.^97,98^ Although its exact mechanism is unclear, hepatotoxicity is suspected to involve the formation of reactive metabolites.^99^ Propiconazole, a triazole fungicide with limited human data, has induced hepatotoxicity and liver tumors in mouse studies, with a higher incidence reported in males.^100,101^

### Quartile-Based Classifications of Toxicity Potential

Model interpretability was assessed by stratifying compounds into quartiles (Q1–Q4) based on normalized *Tox* scores for each endpoint (Figure 5). Higher quartiles represented compounds with higher predicted values (i.e., higher toxicity potential). Biological interpretability was quantified by assessing enrichment of statistically significant pathway perturbations within higher quartiles and their concordance with known mechanistic pathways associated with each toxicity endpoint. Most compounds exhibited low to moderate *Tox* scores; c in Q4 showed markedly higher scores and greater predicted activity in pathways statistically linked to AOs. This quartile-based classification scoring structure enabled comparative evaluation of predicted toxicity across chemicals and supports potential regulatory applications. For example, compounds with acute systemic toxicity were classified using the United Nations Globally Harmonized System of Classification and Labeling of Chemicals (GHS), illustrating how modeled *Tox* scores can complement established hazard classification systems derived from pathway-level predictions.

**Figure 5:**
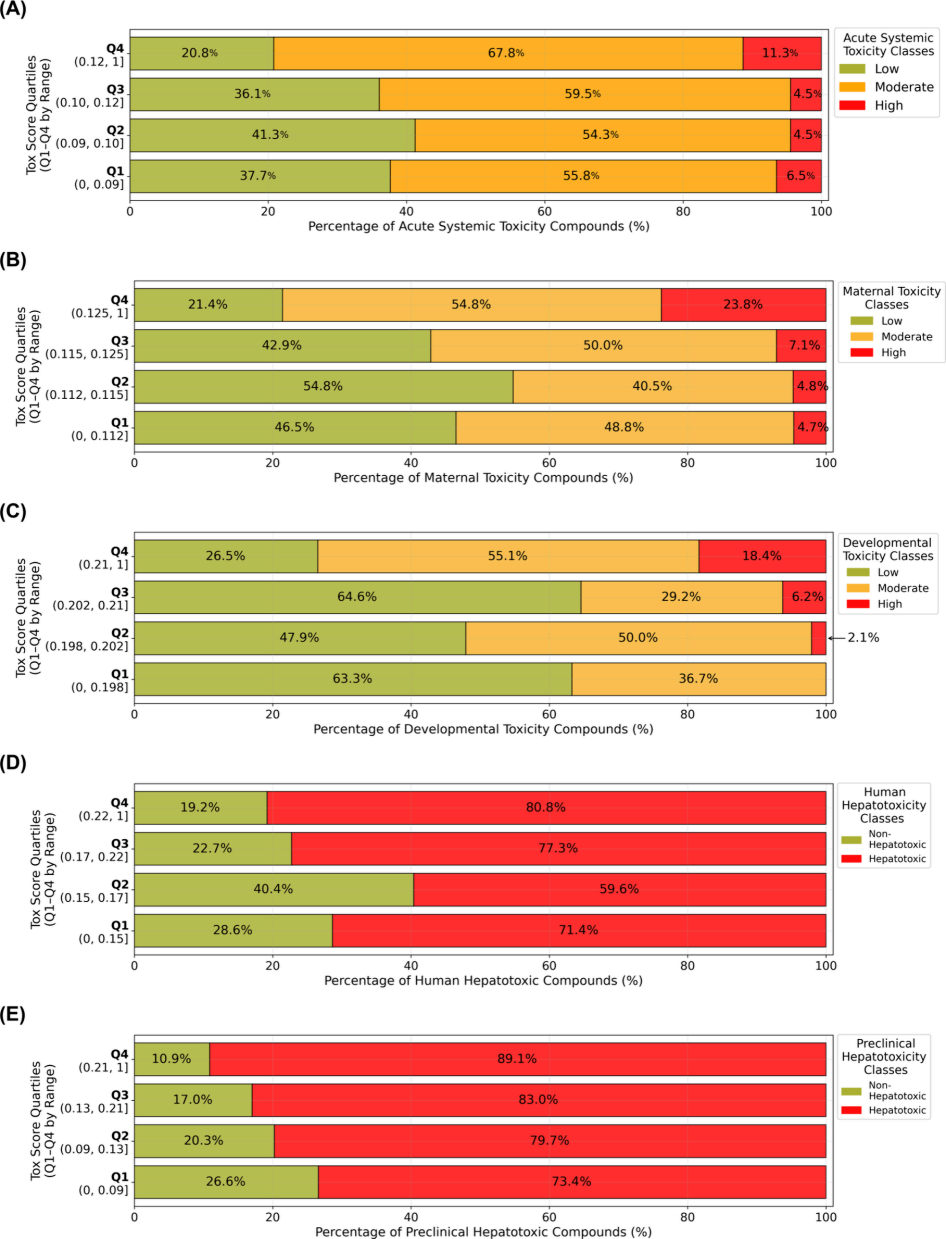
Toxicity class distributions across toxicity score (*Tox*) quantiles. (A) Acute LD_50_, (B) maternal toxicity (NOAEL), (C) prenatal developmental toxicity (NOAEL), (D) human hepatotoxicity, and (E) preclinical hepatotoxicity are shown. Compounds were ranked by normalized Tox scores and divided into quartiles (Q1–Q4). For continuous endpoints, compounds were classified as low (green), moderate (orange), or high (red) toxicity. For binary endpoints, compounds were classified as nonhepatotoxic (green) or hepatotoxic (red). Toxicity class percentages within each quartile are displayed for each endpoint.

For acute systemic toxicity, LD_50_ values were available for 2153 compounds, which were classified according to the GHS criteria: low toxicity (LD_50_ > 2000 mg/kg), moderate toxicity (50 mg/kg < LD_50_ ≤ 2000 mg/kg), and high toxicity (LD_50_ ≤ 50 mg/kg) (Figure 5A).^102^ The *Tox* score quartiles aligned closely with GHS classifications. Most compounds (59.4%, *n* = 1278) were classified as moderately toxic, followed by low-toxicity compounds (*n* = 731) and high-toxicity compounds (*n* = 144) (Supplemental Figure 2A). Within Q4, the proportions of highly toxic (11.5%) and moderately toxic (67.5%) compounds were significantly greater than in Q1, Q2, and Q3, whereas the proportion of low-toxicity compounds was the lowest (21%). Chemical clustering analysis (examples shown in Figure 4A) revealed that compounds in the highest toxicity quartile (Q4) shared structural scaffolds linked to specific toxicity mechanisms. For instance, sulfotep (CAS 3689-24-5), ethion (CAS 563-12-2), and ethoprophos (CAS 13194-48-4) were grouped into Cluster 98 in Figure 4A and consisted of oxygen- and phosphorus-containing chemicals (Figure 4A). These compounds featured dialkyl phosphate moieties and hydrolysis-derived substructures associated with AChE inhibition, the well-known mechanism of acute toxicity.^103,104^ These structural motifs elucidated the high acute systemic toxicity effects (i.e., low LD_50_) observed within this cluster.

Maternal and developmental toxicity were evaluated for 169 and 194 compounds, respectively (Figure 5B,C). Classification thresholds were derived from percentiles of NOAEL distributions from van Ravenzwaay et al., based on threshold of toxicological concern assessments.^79^ Compounds were categorized into low toxicity (NOAEL > 1000 mg/kg bw/day), moderate toxicity (15 mg/kg bw/day < NOAEL ≤ 300 mg/kg bw/day), and high toxicity (NOAEL ≤ 15 mg/kg bw/day) (Figure 5B,C). For maternal toxicity, 70 compounds were classified as low toxicity, 82 as moderate toxicity, and 17 as high toxicity (Supplemental Figure 2B). For developmental toxicity, 98 compounds were classified as low toxicity, 83 as moderate toxicity, and 13 as high toxicity (Supplemental Figure 2C). Across both endpoints, low-toxicity classifications generally decreased from Q1 to Q4, while moderate-toxicity classifications increased. (Figure 5B,C). This monotonic trend between quartile rank and NOAEL-based severity supports the model’s ability to reflect graded toxicological outcomes consistent with empirical data.

For human and preclinical hepatotoxicity, a greater proportion of compounds were classified as hepatotoxic compared to acute and developmental endpoints (Figure 5, Supplemental Figure 2). In the human data set, 220 compounds were classified as nonhepatotoxic, and 573 were hepatotoxic. Human hepatotoxic classifications increased from Q1 to Q4, while nonhepatotoxic classifications decreased (Figure 5D, Supplemental Figure 2D). In the preclinical data set, 233 compounds were classified as nonhepatotoxic and 1013 as hepatotoxic. A similar trend was observed, with hepatotoxic compounds increasing across quartiles and a corresponding decrease in nonhepatotoxic compounds (Figure 5E, Supplemental Figure 2E).

### Integrating Exposure into Chemical Risk Assessments

*C*_ss_ estimates were incorporated into chemical assessments at the assay (*Assay*), protein (*Prot*), and pathway (*Path*) levels to evaluate modeled bioactivity relevant to predicted systemic conditions. *C*_ss_ results were available for 567 compounds in this study, representing approximately 15% of those with valid toxicity predictions (excluding controls). The following case studies illustrate how modeled activity and exposure estimates were jointly assessed for chemical risk characterization (Figure 6).

**Figure 6:**
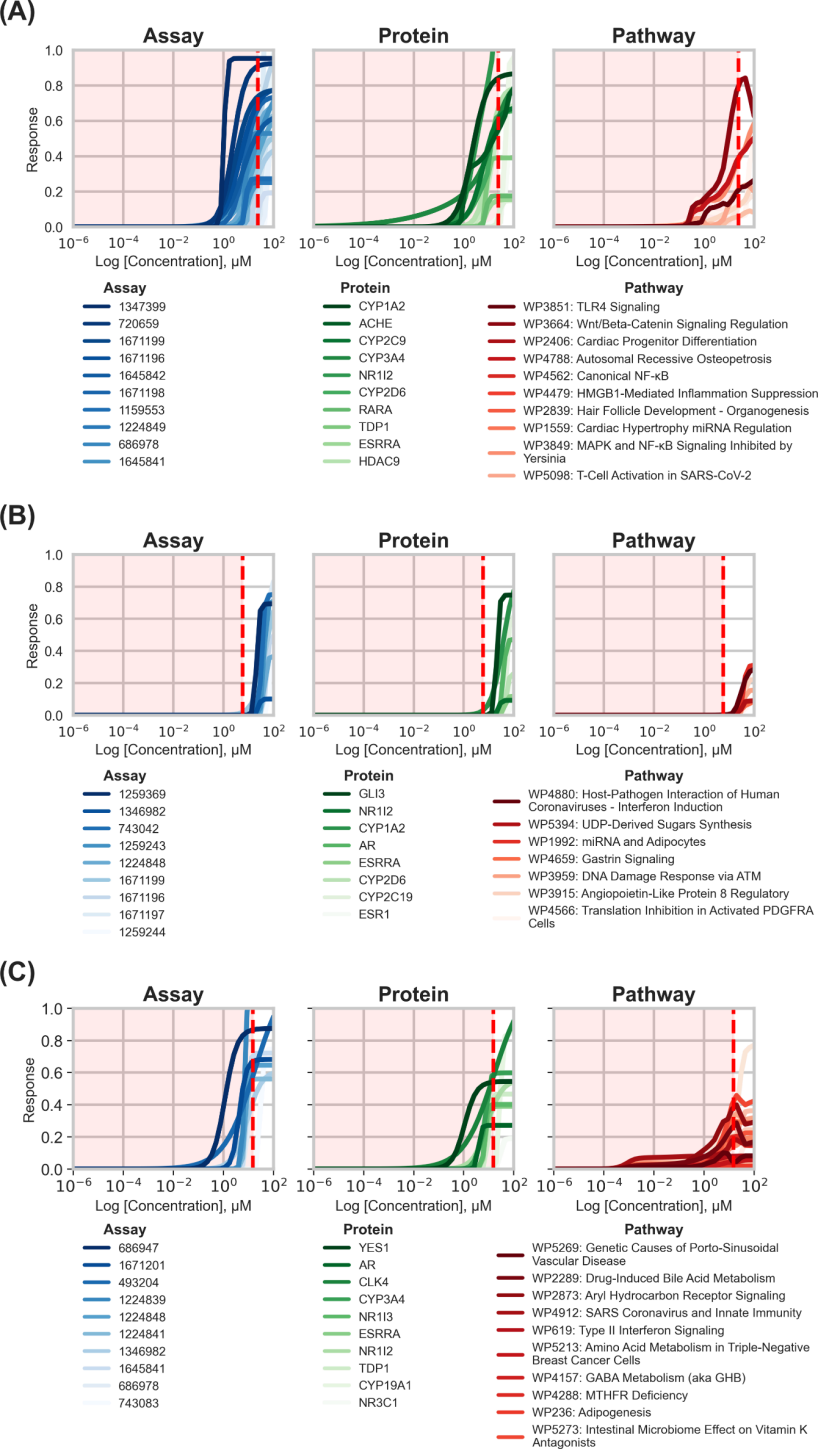
Model concentration–response profiles for coumaphos (A), caffeine (B), and erlotinib (C) at *Assay* (blue, left), *Prot* (green, middle), and *Path* (red, right) layers. Curves are ranked by modeled EC_50_ potency, with the most potent responses plotted at the top of each panel. The red dashed line indicates the estimated log *C*_ss_ value for each compound (1.37 μM for coumaphos, 0.76 μM for caffeine, and 1.17 μM for erlotinib), corresponding to 23.46, 5.78, and 14.96 μM, respectively. The shaded area in each plot indicates modeled concentrations achievable under *in vivo* exposure conditions (i.e., concentrations below *C*_ss_). Pathway-level targets are labeled using WikiPathways (WP) identifiers. Legends display the top 10 most potent targets per layer, whereas all modeled curves are shown. Curves were generated from Hill model fits using normalized HTS data, with an EC_50_ value derived for each compound–target pair.

Coumaphos (CAS 56-72-4) is an organophosphate insecticide that inhibits AChE, disrupting neural signal transmission through cholinergic overstimulation. It received a *Tox* score of 0.29 and was placed in Q4 of the acute systemic toxicity distribution. The estimated *C*_ss_ was 23.46 μM, exceeding the majority of modeled EC_50_ values across all biological levels (*Assay* median: 20.64 μM, *Prot* median: 12.61 μM, *Path* median: 6.43 μM). EC_50_ values of this compound ranged from 1.23 to 4.88 × 10^7^ μM across 25 assays, 2.57 to 5.93 × 10^7^ μM across 14 proteins, and 4.78 to 1.48 × 10^7^ μM across 14 pathways (Figure 6A). The most potent *Assay* responses corresponded to its mechanism of action, AChE inhibition (AID 1347399). Additional activity was observed for pregnane X receptor (PXR) activation (AID 720659), and CYP1A2 and CYP2D6 inhibition (AIDs 1671199, 1671196, respectively), suggesting potential interference with xenobiotic metabolism. At the *Path* level, coumaphos perturbed the TLR4-signaling (WP3851) and Wnt/β-catenin signaling regulation (WP3664), pathways associated with inflammatory and developmental responses.^87,107^ The high *C*_ss_ value relative to modeled EC_50_ values indicates that coumaphos can achieve effective *in vivo* concentrations sufficient to elicit toxicity, consistent with its classification as an acute systemic toxicant.

Caffeine (CAS 58-08-2), a natural central nervous system stimulant, received a *Tox* score of 0.23 and was ranked in Q4 for developmental toxicity (Figure 6B). The estimated *C*_ss_ was 5.78 μM, below all EC_50_ values across biological levels. EC_50_ values for this compound ranged from 21.09 to 82.82 μM across nine assays, 20.77–82.82 μM across eight proteins, and 31.03–49.55 μM across seven pathways. Caffeine modulated several protein/gene targets *in vitro*, including GLI3, NR1I2, AR, ESSRA, ESR1, which are implicated in developmental and endocrine signaling.^108–111^ However, estimated systemic exposure did not exceed modeled effect concentrations for any layer. As a result, caffeine was not prioritized as a developmental toxicant under the model framework because of exposure was insufficient to elicit the predicted bioactivity, with responses occurring at concentrations above *C*_ss_).

Erlotinib (CAS 183321-74-6) is a tyrosine kinase inhibitor used in treating cancers (Figure 6C).^112^ It received a *Tox* score of 0.26 and was classified in Q4 for preclinical hepatotoxicity. The estimated *C*_ss_ was 14.96 μM, exceeding the median EC_50_ values at all biological levels of the model (*Assay*: 11.97 μM, *Prot*: 11.47 μM, *Path*: 9.11 μM). EC_50_ values ranged from 1.32 to 174 μM for 10 assays, from 1.22 to 162.9 μM for 10 proteins, and from 1.42 to 247.6 μM for 23 pathways. The most potent assay responses included inhibitory activity toward YES1 (AID 686947) and CYP3A4 (AID 1671201), linking disrupted survival signaling with reduced hepatic clearance, a profile often associated with compromised hepatic responses to chemical stress. The *Prot* level consisted of YES1, CYP3A4, and CYP19A1, suggesting potential disruption of hepatic enzyme function. At the *Path* level, erlotinib perturbed the aspirin–miRNA interaction pathway (WP4707), which regulates NF-κB-mediated apoptotic signaling in hepatocytes.^113^ With sufficient exposure to induce relevant *in vivo* toxicity pathways (i.e., *C*_ss_ value higher or comparable to modeled EC_50_ values), erlotinib should be classified as a hepatotoxicant, consistent with preclinical evidence of erlotinib-induced hepatotoxicity.^114–116^

This study integrated concentration-dependent responses from diverse HTS platforms into a unified hierarchical architecture that maps assay-level data to protein targets and aggregates them into pathway-level perturbations. The resulting structure supports a stratified representation of compound bioactivity and quantifies mechanistic profiles relevant to *in vivo* toxicity endpoints, including acute systemic, developmental, and hepatic outcomes. Unlike structure-based or single-endpoint modeling strategies, the framework incorporates heterogeneous toxicity data across multiple biological scales. Concentration–response data are propagated through protein and pathway layers, generating reproducible summaries of bioactivities derived from heterogeneous public HTS data. These scores can be directly linked to *in vivo* toxicities of interest. This work demonstrates a pathway-informed strategy for organizing and interpreting HTS data to support iterative knowledge building in NAMs, enabling refinement of pathway resources and development of targeted testing strategies for emerging toxicants.

Recent computational toxicology efforts, such as DeepTox, PrOCTOR, and other data-driven modeling studies, have advanced predictive performance through deep learning, multitask classification, and new modeling algorithms.^53,117,118^ However, these models primarily focus on prioritizing statistical accuracy within single-source training data sets and provide limited mechanistic transparency. In contrast, the hierarchical mechanistic framework presented here explicitly links concentration–response profiles to protein targets, biological pathways, and toxicity endpoints. This organization enables mechanistic interpretation of model outputs, promotes knowledge generation on emerging toxicity mechanisms, and facilitates the integration of heterogeneous HTS data sets across biological levels.

This hierarchical modeling framework quantitatively links *in vitro* mechanistic bioactivity with *in vivo* toxicity outcomes. For chemical prioritization, compounds can be ranked and grouped by pathway-level perturbation scores and predicted toxicity potential for further evaluation. In risk assessment, modeled pathway perturbations and *Tox* scores can be interpreted alongside exposure estimates (*C*_ss_ values) to determine whether observed bioactivity is likely achievable *in vivo* under realistic exposure conditions. In early-phase drug discovery, this framework can identify potential toxicity liabilities by connecting compound bioactivity with established AOPs, thereby informing structure–activity optimization prior to animal and clinical testing. Collectively, these applications demonstrate how structured integration of HTS data within a biologically layered modeling framework enhances the interpretability, transparency, and regulatory usability of computational toxicology models.

The proposed hierarchical modeling framework establishes a robust foundation for NAM-based toxicity modeling byintegrating biological mechanisms and exposure considerations, with promising applicability for formal regulatory adoption. This modeling framework contributes two main advancements to modern computational toxicology. First, it introduces a biologically structured, concentration–response model that processes multisource HTS data into interpretable pathway-informed toxicity predictions, which can be viewed as mechanistic toxicity evaluations of chemical toxicities. Second, it is built for prospective use as a NAM, satisfying essential criteria including nonanimal design, scalability, mechanistic interpretability, and compatibility with TK modeling. These features address the urgent needs of computational toxicology by associating modeled biological perturbations with measured toxicity outcomes, resulting in reproducible and adaptable modeling tools for new data inputs and structured for deployment across public data sets.

## Supplementary Material





## Notes

All genes with one or more associated HTS assays containing concentration–response data (Table S1) and all statistically significant pathways associated with toxicity endpoints from pathway–toxicity correlations (Table S2) (XLSX)
Correlations between modeled pathway scores (*path)* and *in vivo* toxicity outcomes (Figure S1) and ranked compounds by normalized toxicity scores (*Tox*) and colored by *in vivo* toxicity classifications (Figure S2) (PDF)
