# Supplementary material for: Hierarchical Mechanistic Modeling of Complex Toxicity Endpoints from Public Concentration–Response Data
Source: Environ Sci Technol. 2026 Jan 13;60(3):2539–55. doi: 10.1021/acs.est.5c09326 (PMC12854766; doi:10.1021/acs.est.5c09326)
Supplement: Supplementary file 2 [file es5c09326_si_002.pdf]

Supporting Information

Hierarchical Mechanistic Modeling of Complex Toxicity Endpoints from Public Concentration-Response Data

Elena Chung[1,2], Daniel P. Russo[2], Lauren M. Aleksunes[3], Genoa R. Warner[4], Hao Zhu[1,2]*

[1]Department of Chemistry and Biochemistry, Rowan University, Glassboro, New Jersey 08028, United States

[2]Center for Biomedical Informatics and Genomics, School of Medicine, Tulane University, New Orleans, Louisiana 70112, United States

[3]Department of Pharmacology and Toxicology, Ernest Mario School of Pharmacy, Rutgers University, Piscataway, New Jersey 08854, United States

[4]Department of Chemistry and Environmental Science, College of Science and Liberal Arts, New Jersey Institute of Technology, Newark, New Jersey 07103, United States

Corresponding Author

*Hao Zhu, Division of Biomedical Informatics and Genomics, Tulane University, 1430 Tulane Avenue, New Orleans, Louisiana 70112; Telephone: (504) 988-3443; orcid.org/0000-0002-3559-6129, Email: hzhu10@tulane.edu

Table of Contents

Supplemental Figure 1. Correlations between modeled pathway scores (*Path)* and *in vivo* toxicity outcomes. (A) Scatter plots show correlations between *Path* scores and continuous toxicity endpoints: acute systemic toxicity (LD_{50}, top), maternal toxicity (NOAEL, middle), and developmental toxicity (NOAEL, bottom). Toxicity values were $-\log_{10}$-transformed. Pathways were retained if data were available for more than 30 compounds, and linear regression was performed for pathways with at least 15 compounds. Regression models report the coefficient of determination (R^2) values, mean absolute error (MAE), and slope p values. The red-shaded region denotes the 95% confidence interval (CI) of the regression line; the blue-shaded region denotes the 95% prediction interval. (B) Boxplots compare *Path* scores between non-hepatotoxic (blue) and hepatotoxic (red) compounds for human hepatotoxicity (top) and preclinical hepatotoxicity (bottom). Statistical significance was assessed using the Brunner-Munzel test (for distributions with tied ranks) or the Wilcoxon Rank-Sum test. Each plot reports the rank-biserial correlation coefficient (r_{rb}), p-value, and 95% confidence intervals.

Supplemental Figure 2. Ranked compounds by normalized toxicity scores (*Tox*) and colored by *in vivo* toxicity classifications. (A) Acute systemic toxicity (LD_{50}) is classified as high toxicity (red), moderate toxicity (orange), or low toxicity (green). (B) Maternal toxicity and (C) prenatal developmental toxicity are classified similarly using NOAEL thresholds. (D) Human hepatotoxicity and (E) preclinical hepatotoxicity are classified as hepatotoxic (red) or non-hepatotoxic (green) according to binary *in vivo* outcomes. Index represents compounds sorted by decreasing *Tox* scores within each endpoint.

Additional File: Excel spreadsheet containing Supplementary Tables 1 and 2.

Supplemental Material

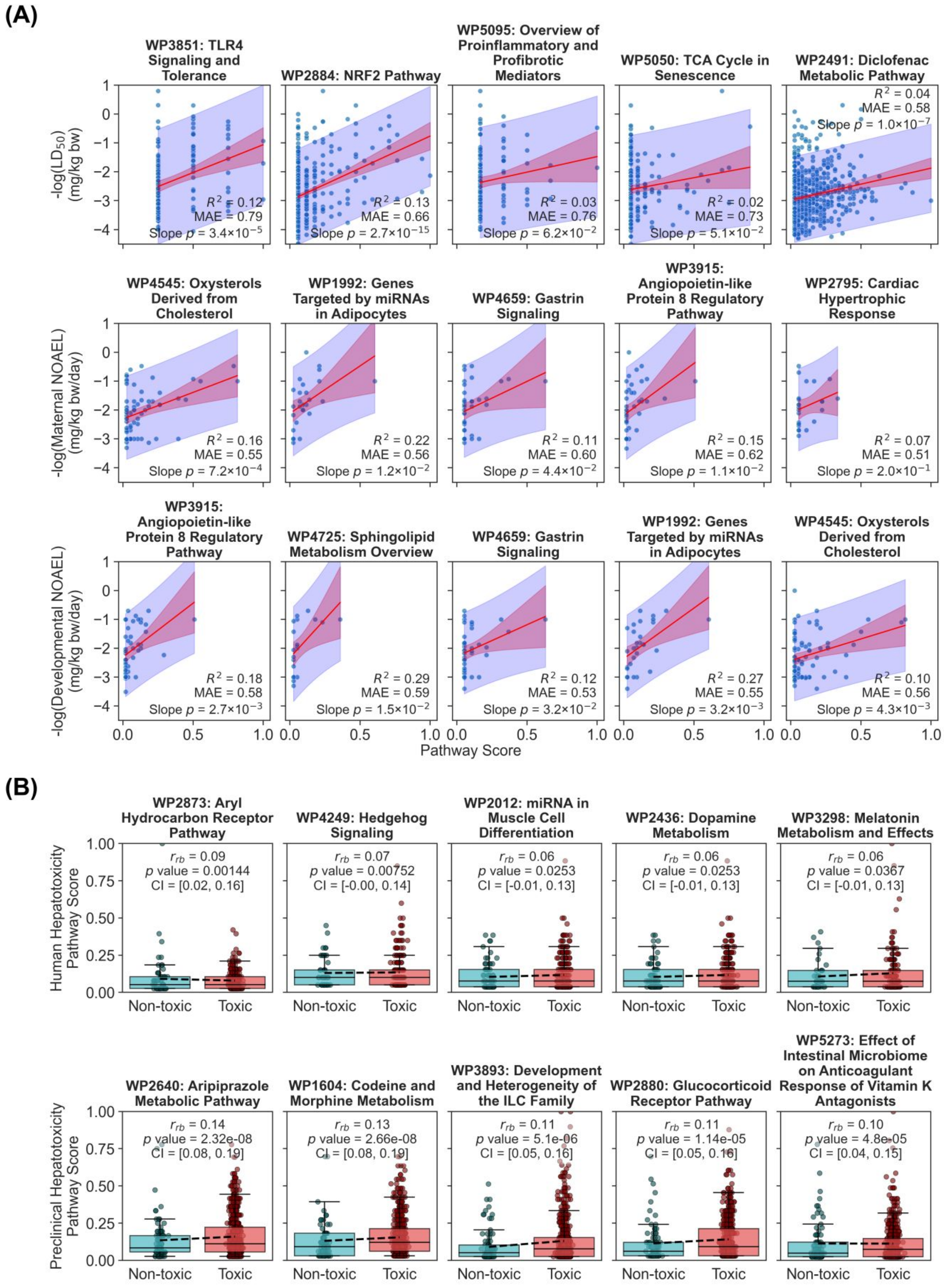

Supplemental Figure 1. Correlations between modeled pathway scores (*Path)* and *in vivo* toxicity outcomes. (A) Scatter plots show correlations between *Path* scores and continuous toxicity endpoints: acute systemic toxicity (LD_{50}, top), maternal toxicity (NOAEL, middle), and developmental toxicity (NOAEL, bottom). Toxicity values were $-\log_{10}$-transformed. Pathways were retained if data were available for more than 30 compounds, and linear regression was performed for pathways with at least 15 compounds. Regression models report the coefficient of determination (R^2) values, mean absolute error (MAE), and slope p values. The red-shaded region denotes the 95% confidence interval (CI) of the regression line; the blue-shaded region denotes the 95% prediction interval. (B) Boxplots compare *Path* scores between non-hepatotoxic (blue) and hepatotoxic (red) compounds for human hepatotoxicity (top) and preclinical hepatotoxicity (bottom). Statistical significance was assessed using the Brunner-Munzel test (for distributions with tied ranks) or the Wilcoxon Rank-Sum test. Each plot reports the rank-biserial correlation coefficient (r_{rb}), p-value, and 95% confidence intervals.

(A)

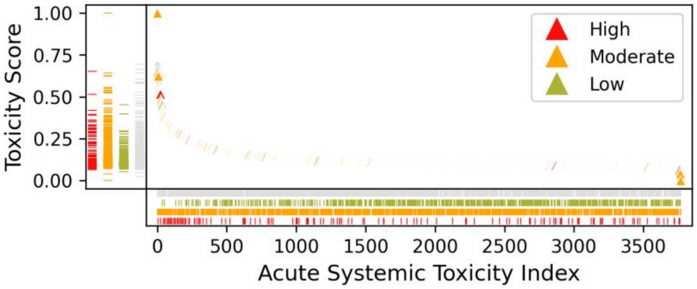
Toxicity Score
1.00
0.75
0.50
0.25
0.00
High
Moderate
Low
0
500
1000
1500
2000
2500
3000
3500
Acute Systemic Toxicity Index

(B)

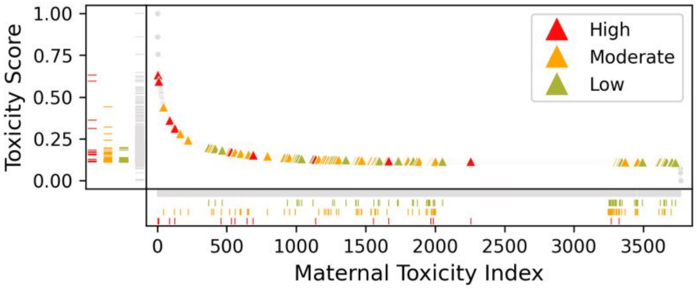
Toxicity Score
1.00
0.75
0.50
0.25
0.00
High
Moderate
Low
0
500
1000
1500
2000
2500
3000
3500
Maternal Toxicity Index

(C)

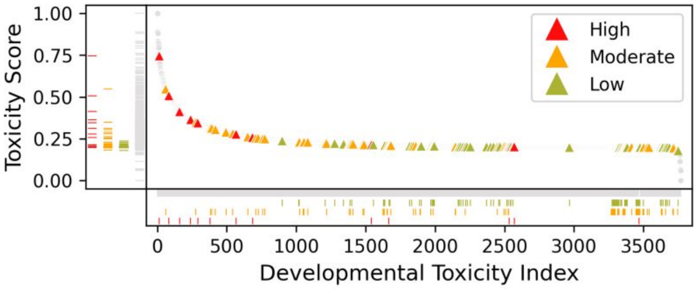
Toxicity Score
1.00
0.75
0.50
0.25
0.00
High
Moderate
Low
0
500
1000
1500
2000
2500
3000
3500
Developmental Toxicity Index

(D)

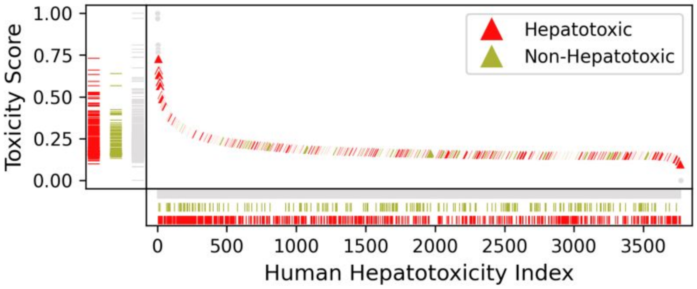
Toxicity Score
1.00
0.75
0.50
0.25
0.00
Hepatotoxic
Non-Hepatotoxic
0
500
1000
1500
2000
2500
3000
3500
Human Hepatotoxicity Index

(E)

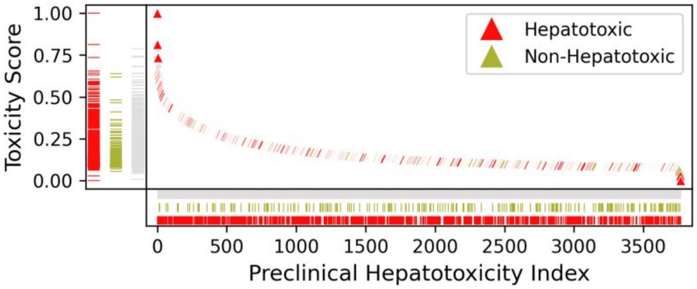
Toxicity Score
1.00
0.75
0.50
0.25
0.00
Hepatotoxic
Non-Hepatotoxic
0
500
1000
1500
2000
2500
3000
3500
Preclinical Hepatotoxicity Index

Supplemental Figure 2. Ranked compounds by normalized toxicity scores (*Tox*) and colored by *in vivo* toxicity classifications. (A) Acute systemic toxicity (LD_{50}) is classified as high toxicity (red), moderate toxicity (orange), or low toxicity (green). (B) Maternal toxicity and (C) prenatal developmental toxicity are classified similarly using NOAEL thresholds. (D) Human hepatotoxicity and (E) preclinical hepatotoxicity are classified as hepatotoxic (red) or non-hepatotoxic (green) according to binary *in vivo* outcomes. Index represents compounds sorted by decreasing *Tox* scores within each endpoint.
